# High-fat diet prevents adaptive peripartum-associated adrenal gland plasticity and anxiolysis

**DOI:** 10.1038/srep14821

**Published:** 2015-10-07

**Authors:** Clara V. Perani, Inga D. Neumann, Stefan O. Reber, David A. Slattery

**Affiliations:** 1 Department of Behavioural and Molecular Neurobiology, University of Regensburg, Regensburg, Germany; 2 Department of Obstetrics and Fetal medicine, Laboratory for Experimental Feto-Maternal Medicine, University Medical Center Hamburg-Eppendorf, Hamburg, Germany; 3 Laboratory for Molecular Psychosomatics, Clinic for Psychosomatic Medicine and Psychotherapy, University of Ulm, Ulm, Germany

## Abstract

Maternal obesity is associated with lower basal plasma cortisol levels and increased risk of postpartum psychiatric disorders. Given that both obesity and the peripartum period are characterized by an imbalance between adrenocorticotropic hormone (ACTH) and cortisol, we hypothesized that the adrenal glands undergo peripartum-associated plasticity and that such changes would be prevented by a high-fat diet (HFD). Here, we demonstrate substantial peripartum adrenal gland plasticity in the pathways involved in cholesterol supply for steroidogenesis in female rats. In detail, the receptors involved in plasma lipid uptake, low density lipoprotein (LDL) receptor (LDLR) and scavenger receptor class B type 1 (SRB1), are elevated, intra-adrenal cholesterol stores are depleted, and a key enzyme in *de novo* cholesterol synthesis, hydroxymethylglutaryl coenzyme A reductase (HMGCR), is downregulated; particularly at mid-lactation. HFD prevented the lactation-associated anxiolysis, basal hypercorticism, and exaggerated the corticosterone response to ACTH. Moreover, we show that HFD prevented the downregulation of adrenal cholesterol stores and HMGCR expression, and LDLR upregulation at mid-lactation. These findings show that the adrenal gland is an important regulator of peripartum-associated HPA axis plasticity and that HFD has maladaptive consequences for the mother, partly by preventing these neuroendocrine and also behavioural changes.

Obesity is a growing public health concern in modern societies^1^,^2^, and maternal obesity has been implicated in an increased risk of postpartum anxiety and depression^3^,^4^,^5^. However the mechanism underlying this increased susceptibility is unclear. A candidate system is the hypothalamic-pituitary-adrenal (HPA) axis, which undergoes substantial plasticity across the peripartum period. Thus, pregnancy and lactation are associated with increased basal glucocorticoid levels (hypercorticism; cortisol in humans; corticosterone in rats and mice) and a concurrent hypo-response to acute stressors^6^,^7^,^8^,^9^,^10^. These adaptations are thought to be an evolutionary mechanism to meet the enhanced energetic demands of the mother and to protect the offspring from high glucocorticoid levels. Moreover, it is speculated that these adaptations contribute to the increased calmness and decreased anxiety that are characteristic of the period^11^,^12^. Recently, it has been shown that obese mothers lack this basal plasma hypercorticism^13^, however, whether interplay between maternal obesity and peripartum HPA axis adaptation exists, and contributes to the increased risk of postpartum psychiatric disorders is currently unknown.

Pregnancy is also associated with increased basal circulating cortisol/corticosterone without changes in its main secretagogue, adrenocorticotropic hormone (ACTH), which normalizes during lactation^8^,^14^. A possible reason underlying this is the peripartum-associated increase in circulating cholesterol; the substrate for adrenal steroid hormone synthesis^15^. Specifically, the adrenal gland sources cholesterol from the plasma *via* low density lipoprotein (LDL) receptor (LDLR)-mediated endocytosis and selective up-take *via* scavenger receptor class B type 1 (SRB1). In addition, cholesterol can be locally *de novo* synthesized, relying on hydroxymethylglutaryl coenzyme A reductase (HMGCR) activity, or recruited from cholesterol esters stored in adrenal lipid droplets^16^. Such intra-adrenal cholesterol esters are synthesized by the enzyme acetyl-coenzyme A acetyltransferase (ACAT) and hydrolyzed to free cholesterol by the enzyme hormone-sensitive lipase (HSL), and are believed to represent the preferential cholesterol source recruited by ACTH-induced (e.g. stress-induced) steroidogenesis^16^,^17^. Given that (i) the peripartum period is associated with hyperlipidemia, (ii) the lipid cholesterol is required for steroidogenesis^17^ and (iii) changes in cholesterol availability have been linked to altered HPA axis function^18^,^19^, we hypothesized that the adrenal pathways involved in free cholesterol supply for steroidogenesis would show peripartum-related plasticity. Having shown this, we next assessed whether high-fat diet (HFD) intake functionally impinged on these alterations and *in vivo* HPA axis (re)activity at mid-lactation, since this was found to be the time of greatest adrenal plasticity.

## Results

### Adrenal glands display plasticity across the peripartum period

**Figure 1:**
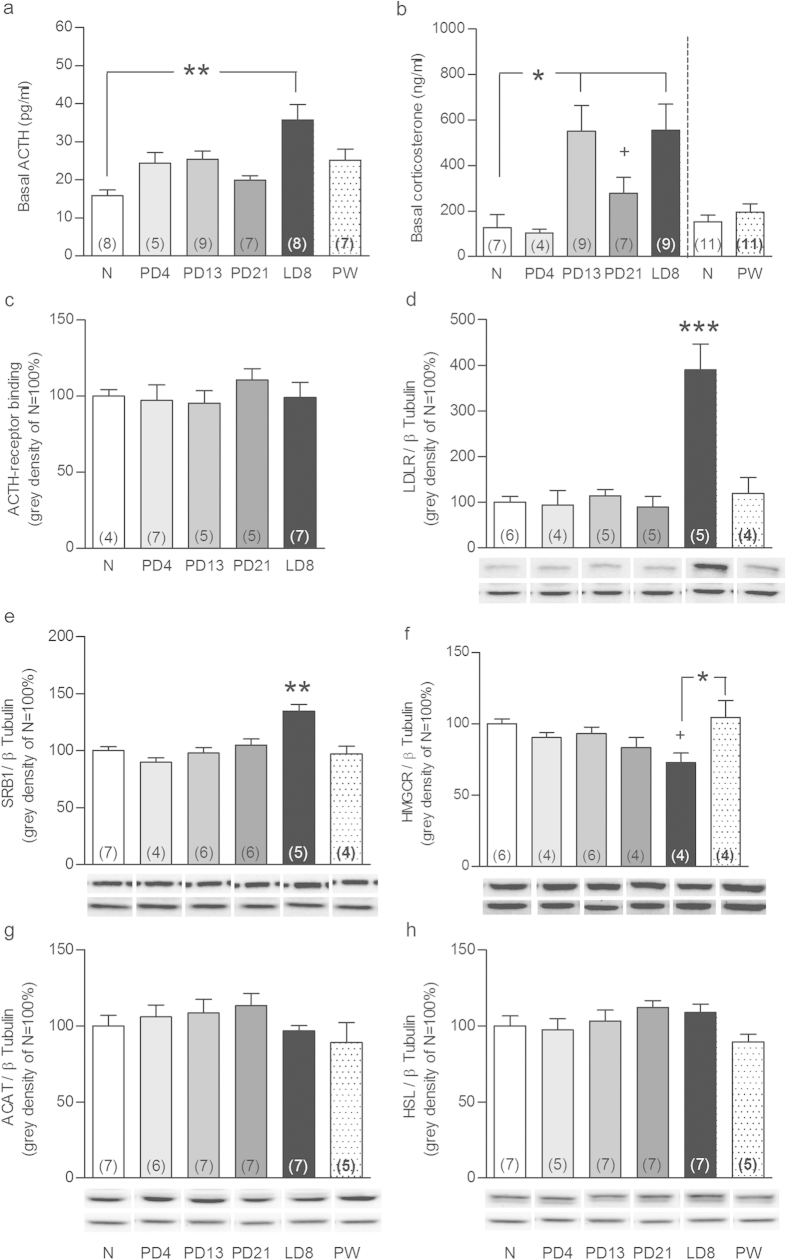
Reproductive state affects basal ACTH and corticosterone levels and the adrenal glands. ACTH (**a**) and corticosterone (**b**) levels in trunk blood, adrenal ACTH-receptor binding (**c**), LDLR (**d**), SRB1 (**e**), HMGCR (f), ACAT (**g**) and HSL (**h**) protein expression in nulliparous rats at diestrous/metestrous (N) and animals at pregnancy day (PD) 4, PD13, PD21, lactation day (LD) 8 and 4 weeks post weaning post-weaning (PW) are presented. Adrenal ACTH-receptor binding and proteins are expressed as % relative to N rats and protein bands were normalized to β Tubulin in the same membrane. Representative blots are shown below the corresponding graphs (upper band) together with the loading control β Tubulin (lower band). Full-length blots are presented in the Supplementary Unedited Gels Fig. 1 file. Data represent mean + SEM (numbers in parenthesis are the n numbers). Statistical significance was determined using a one-way ANOVA followed by Bonferroni *post-hoc* test or Mann Whitney U test. ***P < 0.001, **P < 0.01 and *P < 0.05 vs. all the groups unless vs. a specific group when indicated; ^+^P < 0.05 vs. N females after Mann Whitney U test analysis.

To determine whether the adrenal glands play a role in HPA axis plasticity across the peripartum period, we collected plasma and adrenal glands from nulliparous rats and rats from various points across the peripartum period. We first confirmed that reproductive state affected both plasma ACTH (F_5,44_ = 3.16, P = 0.017) and corticosterone (F_4,35_ = 4.79, P = 0.004). While basal ACTH levels were only increased on lactation day (LD) 8 (P < 0.01; Fig. 1a), corticosterone levels were higher from pregnancy day (PD) 13 to LD8 (P < 0.05; Fig. 1b) compared with nulliparous rats. Separate analysis revealed that plasma corticosterone was higher at PD21 compared with nulliparous females (Mann Whitney U, P < 0.05; Fig. 1b). Corticosterone levels in the 4 week post-weaning (PW) group were assessed in a separate experiment and were not different to nulliparous rats (right panel Fig. 1b). Comparison among nulliparous rats revealed highest corticosterone levels at proestrous with no changes in ACTH (see Supplementary Fig. 1a and b); confirming the transient ACTH-corticosterone imbalance observed across the estrous cycle^20^.

**Figure 2:**
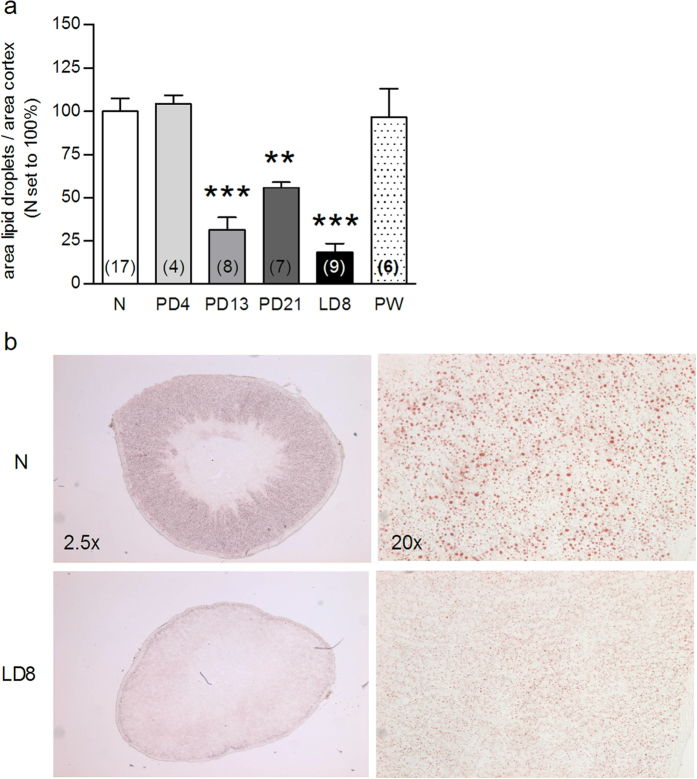
Cortical lipid droplets are markedly depleted in pregnant and lactating rats. Adrenal cortical lipid droplets (**a**) were quantified in nulliparous (N) rats and animals at pregnancy day (PD) 4, PD13, PD21, lactation day (LD) 8 and 4 weeks post-weaning (PW). Representative pictures (**b**) from N and LD8 animals of stained adrenal sections are reported (2.5 and 20× magnification). Data represent mean + SEM (numbers in parenthesis are the n numbers). Statistical significance was determined using a one-way ANOVA followed by Bonferroni *post-hoc* test. ***P  < 0.001 compared with N, PD4 and PW animals; **P < 0.01 vs. N.

Given this dissociation, we next assessed adrenal pathways involved in free cholesterol supply for steroidogenesis to determine, if they are involved in the aforementioned peripartum-associated HPA axis adaptations. Reproductive state did not affect adrenal weight (Supplementary Fig. 2) or ACTH receptor binding (Fig. 1c). In contrast, adrenal protein expression of the two main receptors involved in lipid uptake from the plasma, LDLR (F_5,23_ = 14.4, P < 0.001) and SRB1 (F_5,31_ = 9.06, P < 0.001), was increased at LD8 compared with all other groups (P < 0.001, Fig. 1d and P < 0.01, Fig. 1e, respectively). Protein expression of HMGCR (F_5,22_ = 3.12, P = 0.028), the key enzyme in *de novo* cholesterol synthesis, was reduced at LD8 compared with PW (P < 0.05) and nulliparous rats (Mann Whitney U; P < 0.05; Fig. 1f). Protein levels of ACAT and HSL, the enzymes involved in cholesterol-ester synthesis and breakdown, respectively, were not affected by reproductive state (Fig. 1g, h). Adrenal lipid stores, quantified *via* oil-red lipid staining (F_5,50_ = 19, P < 0.001), were markedly reduced from mid-pregnancy to mid-lactation compared with nulliparous rats (all P < 0.01; Fig. 2a). These effects were predominantly specific to the peripartum period, as all were normalized to nulliparous levels PW (Figs. 1 and 2) and only LDLR expression was altered, and to a lesser extent, across the estrous cycle (Supplementary Fig. 1c–h). Taken together, these findings suggested for the first time that the adrenal gland is an important mediator of HPA axis-related peripartum adaptations.

### HFD alters maternal *in vivo* HPA axis function and anxiety-related behavior

**Figure 3:**
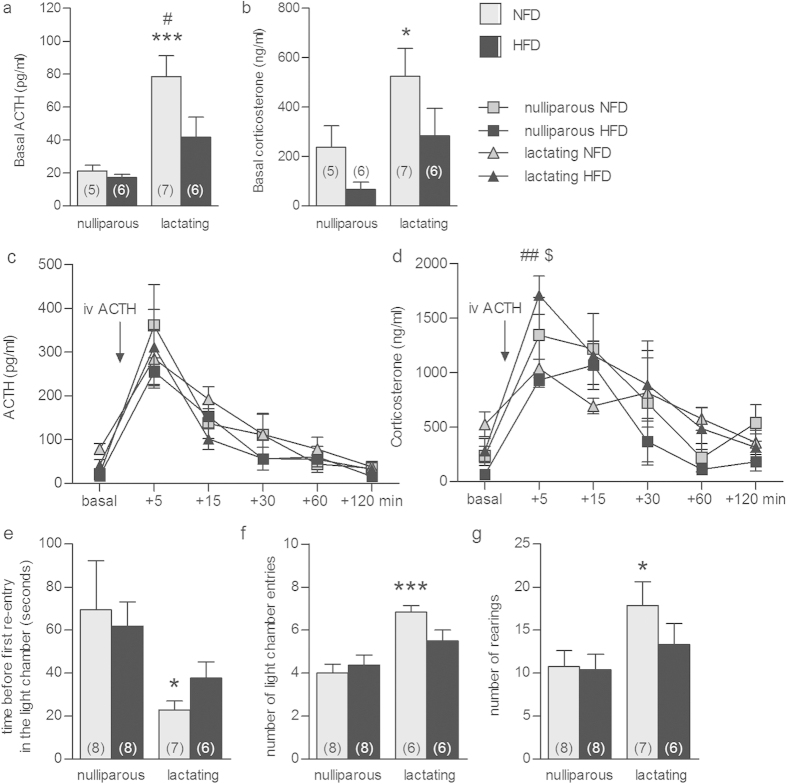
High-fat diet (HFD) prevented the increase in basal ACTH and corticosterone, exaggerated the corticosterone response to ACTH *iv* injection and affected anxiety-related behaviour in lactating animals. Basal ACTH (**a**) and corticosterone (**b**), ACTH (**c**) and corticosterone (**d**) levels before and after ACTH *iv* and light-dark box test parameters (i.e. time before first re-entry in the light chamber (**e**), number of light chamber entries (**f**) and rearings (**g**)) in nulliparous and lactating animals normal fat diet (NFD) or fed on a HFD are represented. Data represent mean + SEM (numbers in parenthesis are the n numbers). Statistical significance was determined using a two-way ANOVA (**a**, **b**, **e**, **f**, **g**) with repeated measures (**c**, **d**) followed by Bonferroni *post-hoc* test. ***P ≤ 0.001 and *P < 0.05 lactating vs. nulliparous NFD; ^##^P < 0.01 and ^#^P < 0.05 lactating NFD vs. lactating HFD; ^$^P ≤ 0.001 lactating HFD vs. nulliparous HFD.

To gain a better understanding of the functional consequence of these peripartum alterations in adrenal gland plasticity, we next assessed the impact of HFD on HPA axis function and anxiety-related behavior at LD8. HFD significantly increased body weight gain in both the nulliparous and peripartum group (factor time x diet: F_10,250_ = 6.36, P < 0.001; normal fat diet (NFD) vs. HFD P < 0.05; Supplementary Fig. 3). We could replicate our findings showing a lactation-associated increase in basal ACTH (F_20,1_ = 17.3, P < 0.001) and corticosterone (F_20,1_ = 6.94, P = 0.016) in NFD compared with nulliparous rats (P < 0.001, Fig. 3a and P < 0.05, Fig. 3b, respectively) and show that HFD (ACTH: F_20,1_ = 4.3, P = 0.05; corticosterone: F_20,1_ = 4.57, P = 0.045) abolished these effects (ACTH: HFD dam vs. NFD dam P < 0.05; Fig. 3a). Moreover, whereas peak ACTH levels obtained 5 min after a stress-equivalent intravenous (*iv*) ACTH injection did not differ among the groups (Fig. 3c), corticosterone levels were affected (factor state × diet: F_1,18_ = 4.94, P = 0.039). In detail, HFD dams displayed an exaggerated corticosterone response compared with NFD dams (P < 0.01) and nulliparous HFD rats (P ≤ 0.001; Fig. 3d) 5 min after *iv* ACTH administration. Importantly, we could also reveal that HFD prevented lactation-associated anxiolysis, but did not affect anxiety-related behavior in nulliparous animals. Specifically, lactation-induced anxiolysis was confirmed given that the reproductive state affected the latency to re-enter the lit chamber of the light-dark box (F_1,25_ = 5.79, P = 0.024; Fig. 3e), the number of lit chamber entries (F_1,24_ = 20.0, P < 0.001; Fig. 3f), and the rearing in the lit chamber (F_1,25_ = 5.17, P = 0.032; Fig. 3g). Indeed, NFD dams re-entered the lit chamber faster (P < 0.05), more often (P < 0.001), and showed increased rearing behavior in the lit compartment (P < 0.05) compared with nulliparous NFD rats without affecting locomotion. In contrast, no difference in these parameters were observed in HFD lactating rats. Taken together, these findings reveal that HFD detrimentally affects maternal HPA axis adaptations and anxiolysis.

### HFD alters lactation-associated adrenal plasticity

**Figure 4:**
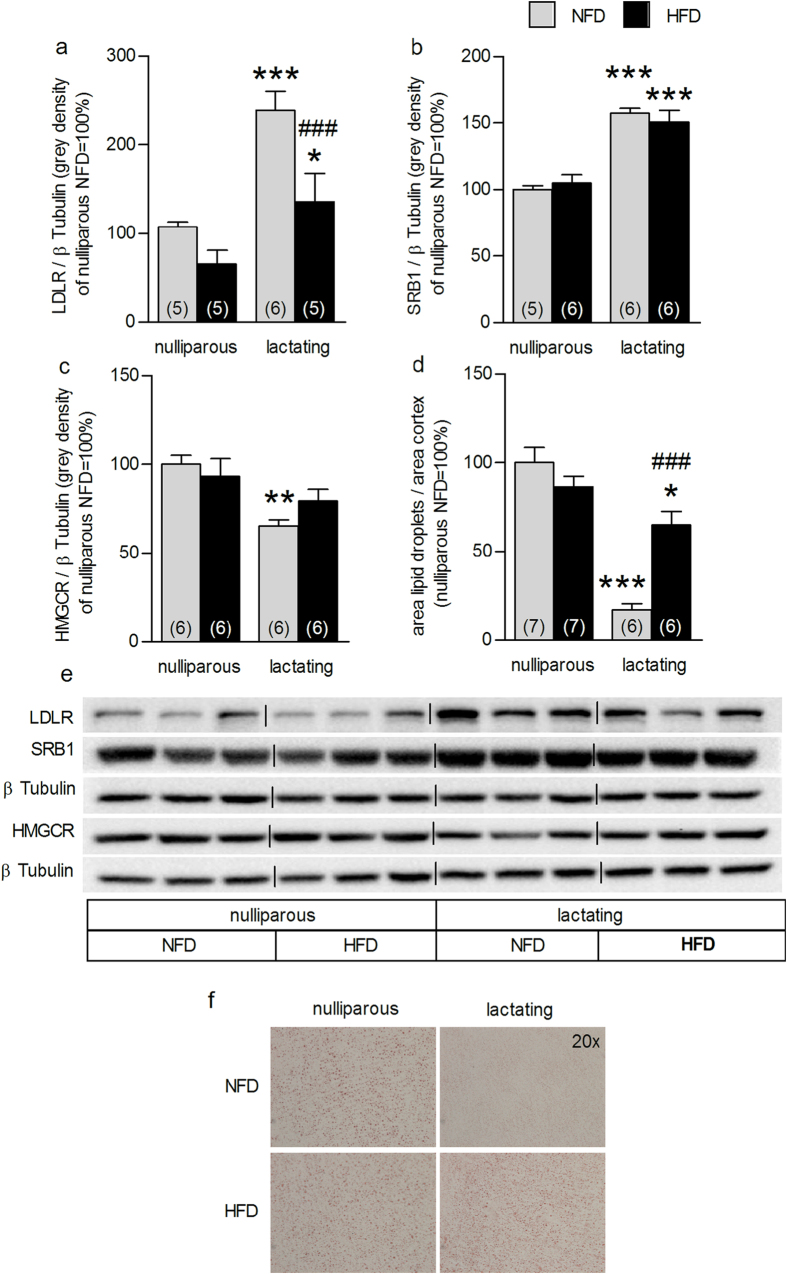
High-fat diet (HFD) feeding impacted on lactation-associated adrenal plasticity. Adrenal LDLR (**a**), SRB1 (**b**), HMGCR (**c**) protein expression and cortical lipid droplets (**d**) in nulliparous and lactating rats normal fat diet (NFD) and fed on a HFD are reported. Data represent mean + SEM (numbers in parenthesis are the n numbers). All protein bands were normalized to β Tubulin in the same membrane. Representative blots (**e**) of the proteins of interest and loading control and pictures (**f**) of stained adrenal sections (20× magnification) are shown. Full-length blots are presented in the Supplementary Unedited Gels Fig. 4 file. Statistical significance was determined using a two-way ANOVA followed by Bonferroni *post-hoc* test. ***P < 0.001, **P < 0.01, *P < 0.05 vs. respective nulliparous group. ^###^P ≤ 0.001 vs. lactating NFD dams.

To determine whether these HFD-induced alterations in HPA axis (re)activity were mirrored at the level of the adrenal gland, we next assessed the impact of HFD on adrenal gland plasticity at LD8. Both reproductive state (F_3,20_ = 26.4, P < 0.001) and diet (F_3, 20_ = 16.4, P = 0.001) affected adrenal LDLR expression (Fig. 4a). Specifically, the lactation-induced increase in LDLR protein was reconfirmed (NFD P < 0.001; HFD P < 0.05), whereas HFD dams showed reduced adrenal LDLR protein expression compared with NFD dams (P ≤ 0.001; Fig. 4a). However, the lactation-associated increase in SRB1 protein expression was not affected by HFD (F_3,22_ = 69.6, P < 0.001, lactating vs nulliparous P < 0.001; Fig. 4b). Also the lactation-induced reduction in HMGCR protein level (F_3,23_ = 5.26, P = 0.002; NFD, P < 0.01, Fig. 4c) was abolished by HFD. Neither reproductive state nor diet affected ACTH-receptor binding, ACAT or HSL adrenal protein expression (see Supplementary Fig. 4a–c). The lactation-associated decrease in adrenal lipid stores (factor state × diet: F_3,25_ = 19.9, P < 0.001; NFD P < 0.001; HFD P < 0.05) was confirmed, but HFD dams showed increased lipid droplets compared with NFD dams (P < 0.001; Fig. 4d, f). These results show that HFD affects the normal adrenal gland plasticity at mid-lactation and thus, presumably contributes to the alterations in *in vivo* HPA axis (re)activity.

## Discussion

In the present study, we report for the first time that the three pathways that lead to free cholesterol supply within the adrenal gland, i.e. up-take from plasma lipoproteins, *de novo* synthesis and intra-adrenal stores mobilization, substantially adapt across the peripartum period. Moreover, the majority of these changes, as well as lactation-associated basal hypercorticism, reduced acute stress responsiveness, and maternal anxiolysis are prevented by exposure to HFD. These changes were specific to the peripartum period as HFD did not affect these parameters in nulliparous rats. Taken together, our findings support a major role of the adrenal gland in the peripartum-associated changes in the HPA axis function and that prevention of adrenal plasticity, such as by obesity, may increase the risk for the development of postpartum psychopathologies such as anxiety.

As an important prerequisite to our hypotheses, we initially recapitulated literature findings revealing the ACTH-corticosterone imbalance across both the estrous cycle and peripartum period^11^,^20^. While we could confirm the increase in corticosterone levels during pregnancy without a concomitant increase in plasma ACTH, we observed an unexpected increase in ACTH in lactation. This increase was observed in both experimental cohorts and from trunk blood and catheter samples suggesting this to be a robust finding. However, the reason for this discrepancy is unknown, although relatively few studies have assessed ACTH levels in lactation (in comparison to nulliparous females). We could additionally reveal that ACTH-receptor binding does not alter across the peripartum period, suggesting that other factors sustain the increased level of steroidogenesis at this time. Indeed, adrenal pathways involved in free cholesterol supply for steroidogenesis substantially adapt across the peripartum period, with maximal plasticity observed in mid-lactation. This was indicated by a depletion of adrenal lipid droplets from PD13 to LD8, which is likely to reflect increased cholesterol-ester mobilization, followed by increased synthesis and secretion of corticosterone into plasma, mediating the known phenomenon of basal hyercorticism. On the other hand, the increase in LDLR and SRB1 and decrease in HMGCR protein expression was only observed at LD8, suggesting a surrogate mechanism to maintain basal hypercorticism after the lipid droplets are depleted. The depletion of adrenal lipid droplets during pregnancy without changes in total protein expression in steroidogenic proteins involved in cholesterol metabolism may be explained by the fact that the activity of HSL is governed by its phosphorylation state^21^. This hypothesis will be explored in future studies. Such changes were relatively specific to the peripartum period, as only LDLR was altered across the estrous cycle, with increased expression found at estrous. Furthermore, all these peripartum changes revert to a nulliparous phenotype four weeks after weaning.

Given that these results suggested a prominent role of changes in the adrenal lipid metabolism in peripartum-associated physiological, and presumably also behavioral, adaptations we next assessed whether HFD exposure would affect maternal behavior and physiology. In keeping with human studies showing that overweight women are at risk for elevated anxiety and depressive symptoms, both during pregnancy^3^ and within the first 14 months postpartum^3^,^4^, we found that lactation-associated anxiolysis was prevented by HFD. Importantly, we could also demonstrate that the lactation-associated basal high corticosterone levels as well as the well-known hypo-responsiveness to acute stressors (here ACTH *iv* injection)^7^,^11^ were abolished in HFD dams. In terms of the latter finding, dissociation of plasma corticosterone between NFD and HFD, but not of plasma ACTH levels, 5 minutes post-injection, clearly indicates the involvement of adrenal-mediated mechanisms. This could be further supported as most of the adrenal changes at mid-lactation were prevented by HFD. Specifically, lipid droplet levels and HMGCR expression were found to be similar to nulliparous levels in HFD dams and the lactation-associated increase in LDLR expression was strongly attenuated by HFD. Further, our data are in agreement with results from a similarly designed human study where *iv* ACTH elicited higher cortisol plasma levels in obese compared with non-obese women^22^.

Adrenal mRNA expression of steroidogenic genes, including Star and Cyp11A1, has been shown to be affected by a 60% HFD in mice^23^ suggesting that HFD might interfere with adrenal function at this level. However, while we found an overall reproductive status effect on Star, but not Cyp11A1, mRNA expression, HFD did not affect these parameters. Our findings suggest that Star mRNA levels are higher during lactation, but no post-hoc comparisons reached significance. Therefore, it is possible that changes downstream of cholesterol supply occur across the peripartum period (Supplementary Data and Table 2), which will be assessed in future studies.

The alterations in behavior and HPA axis function were specific to the postpartum period as HFD did not affect any of these parameters in nulliparous rats. Taken together, these HFD-induced changes indicate an overall increase in adrenal cholesterol availability, which is likely due to the lack of basal hypercorticism and involved in the increased acute stress response detected in HFD dams.

In conclusion, we have demonstrated a crucial role of adrenal gland plasticity for maternal physiology. Indeed, prevention of such plasticity *via* HFD results in altered basal HPA axis function, an exaggerated stress response, and the abolishment of maternal anxiolysis. Thus, since interference with peripartum HPA axis plasticity is known to participate in the etiology of several postpartum disorders, their prevention by maternal obesity may contribute to the increased susceptibility to such disorders in obese women.

## Materials and Methods

### Animals

Female Wistar rats (200 to 250 g, from Charles River, Sulzfeld, Germany) were left undisturbed for one week after arrival under standard group (3–4 rats) housing and environmental (12 h light-12 h dark, lights on at 06:00, 22 ± 1 °C, 60 ± 5% humidity) conditions. Animals were fed a standard diet (normal food - NFD; 10% fat; Ssniff, Germany) throughout each experiment except the HFD studies, where animals received either the NFD or a HFD (45% fat, Research Diets Inc., U^24^) starting two weeks prior to mating until the end of the experiment. All experimental procedures, performed between 08:00–12:00, were approved by the Committee on Animal Health and Care of the local government of the Oberpfalz and complied with international guidelines on ethical use of animals.

### Experimental procedures

#### Estrous cycle-associated adaptations

Nulliparous rats were decapitation (08:00–11:00) after four days of single housing and vaginal smears were collected to delineate the three experimental groups: di-/metestrous, proestrous and estrous; diestrous and metestrous females were pooled given that ACTH and corticosterone levels have already been reported to be similar across these two groups^20^. Trunk blood and adrenal glands were collected and processed (see Methods section). Potential body-side-specific adrenal adaptations^25^ were assessed at first; after showing that no parameter of interest differed between left and right adrenal (see Supplementary Table 1), the estrous cycle investigation was performed in left adrenals.

#### Peripartum-associated adrenal adaptations

Rats were time-mated (see Methods section) to result in the following concurrent rat groups: nulliparous (including di-/metestrous from the estrous cycle experiment), PD4, PD13, PD21, LD8 and 4-weeks PW. Females at proestrous were excluded because the estrous cycle investigation revealed a marked proestrous-associated increase in basal corticosterone (see Supplementary Fig. 1). Left and right adrenal measures were pooled in each group as no side differences were detected for any parameter. Animals were single-housed four days before tissue collection with the exception of LD8 animals, which were single-housed four days before predicted parturition (PD17) to allow undisturbed birth and nursing of the offspring. After weaning, PW dams were group-housed until four days before adrenal glands and trunk blood were collected as previously described^26^(see Methods section).

#### HFD effects on behavior and HPA axis

Nulliparous and LD8 NFD and HFD were tested for anxiety-related behavior in the light-dark box (see Methods section) on LD3 and HPA axis (re)activity on LD8. All animals were single-housed on PD17, or equivalent in nulliparous rats.

#### HFD effects on adrenal plasticity

HFD impact on adrenal plasticity was investigated in nulliparous and LD8 NFD and HFD animals. Trunk blood and adrenal glands were collected and processed (see Methods section). All animals were single-housed on PD17, or equivalent in nulliparous rats.

### Methods

#### Time-mating and verification of pregnancy

In each study, female rats were mated (two to three females per male), and pregnancy was verified by vaginal smears (designated PD0). After parturition, defined as LD0, pups were culled to four females and four males per litter as it has been shown that litter composition can influence maternal behavior^27^.

#### ACTH and corticosterone measurements

Plasma was isolated from trunk blood (5000 rcf, 10 min, 4 °C), stored in aliquots at −20 °C until ACTH and corticosterone were measured *via* commercially available ELISA kits (IBL International GmbH, Germany – corticosterone kit assay range: 1.63–240 nmol/L, ACTH kit assay sensitivity: 0.22 pg/mL).

#### Organ preparation

Each left and right adrenal from the estrous cycle and peripartum-associated adrenal adaptation studies was weighed and cut in half. Afterwards, one half was embedded in protective freezing medium (Tissue-Tek) and the other snap-frozen in liquid nitrogen, before being stored at −80 °C for cryo-cutting or western blot analysis, respectively. After embedding, a series of 16 μm cryo-sections from the mid-part of the adrenal glands, containing both the cortex and medulla, was cut and then thaw-mounted onto pre-coated slides (six sections per slide). Since no side-differences were detected in the experiments described so far, left adrenals from the HFD study were embedded in freezing medium for cryo-sectioning and right adrenals were snap-frozen for protein isolation.

#### Oil red lipid staining

Adrenal cryo-sections were stained with Oil-red-O to quantify adrenal lipid droplet cholesterol stores as previously described^18^,^28^. Six sections per adrenal were stained, photographed, and quantified. Each adrenal section was considered as divided through two imaginary lines, crossing in the middle of the adrenal medulla^18^,^28^. From each area, a microscopic image at 5× magnification was collected using the Leica V4 image acquisition program. Subsequently, one picture from each of the six adrenal slices per animal was analysed and the average, calculated from the six measures, was used for statistical analysis. The oil-red positive area (mm^2^) within the zona fasciculata and zona reticularis and the total area of these two cortical zones (mm^2^) were measured using Leica FW4000 software (Leika Microsystems, Germany), and the ratio between the two was calculated. The adrenal medulla and glomerulosa, both easily distinguishable by eye^29^, were not considered in this analysis as the main adrenal zones involved in corticosterone synthesis are the fasciculata and reticularis^30^.

#### ACTH receptor autoradiography

ACTH receptor autoradiography was performed on six adrenal sections per animal using the linear ACTH receptor antagonist [^125^I]ACTH, (1–39) Tyr23 as tracer (PerkinElmer, US) as previously described^31^. In detail, six adrenal sections per animal were thawed, dried and fixed for two minutes in 0.1% paraformaldehyde at room temperature. After two 10 min washing steps in 50 mM Tris, sections were exposed for 60 minutes to the tracer buffer (50 mM tracer, 10 mM MgCl_2_, 0.1% BSA) and then washed three times in Tris 50 mM, MgCl_2_ 10 mM buffer. Slides were then dipped in water, air-dried and exposed for 90 days to Biomax MR films.

ImageJ 1.47 was used to measure receptor binding, expressed as grey density, and background signal was subtracted to control for non-specific binding.

#### Western blot analysis

Western blot analysis was performed to measure adrenal expression of proteins involved in the supply of free cholesterol as previously described^18^. Protein levels of LDLR and SRB1, HMGCR, ACAT and HSL were analysed. Adrenal proteins were isolated via lysis buffer (50 mM Hepes, 250 mM NaCl, 0,5 mM EDTA, 0,5% Igepal) supplemented with protease inhibitor cocktail and the protein concentration was determined using the Pierce BCA Protein Assay Kit. 25 μg from each protein extract was separated via electrophoresis, performed using a 10% SDS-polyacrylamide gel, and transferred onto a nitrocellulose membrane. TBS/0.1% Tween-20 (TBST, pH 7.6) supplemented with 5% milk powder was used to block the membranes (1 hour at room temperature) and to dilute primary antibodies (Ab). Blots were incubated over night at 4 °C with the specific primary Ab at 1:1000 dilution against LDLR, SRB1 (Abcam, UK), HMGCR, ACAT (Santa Cruz, US), and HSL (Cell Signalling, US). For secondary Ab incubation, membranes were incubated at room temperature for 1 hour with peroxidase-conjugated anti-rabbit IgG (Cell signalling, US) diluted 1:1000 in TBST. Blots were washed with TBST after each Ab-incubation session. All protein bands were normalized against β Tubulin (anti-β Tubulin, Cell Signalling; 1:1000; overnight at 4 °C) in the same membrane for loading. Secondary Ab incubation was performed as described above. Bands were visualized using ECL western blot detection reagents, and images were acquired with the ChemiDoc XRS+ system. Each western blot was run in duplicate and the average of two protein expression values per adrenal was used for statistical analysis.

#### Anxiety-related behaviour

The light-dark box was performed to assess anxiety-related behavior on LD3 or equivalent in nulliparous rats as previously described^32^,^33^. The experimental setup consisted of one lit (40 × 50 cm, 350 lux) and one dark (40 × 30 cm, 70 lux) compartment connected via a small opening (7.5 × 7.5 cm), enabling transition between the compartments across the 5 min test with the rats placed in the lit compartment facing away from the opening. The floors in each compartment were divided into squares (10 × 10 cm). The latency to the first entry in the dark chamber, latency to the first re-entry in the lit chamber, lit chamber entries, and rearings were used to assess anxiety-related behavior. The number of line-crossings were measured as an indicator of locomotor activity^32^.

### Jugular catheter and blood sampling

On LD4 a jugular vein catheter was implanted under isoflurane anesthesia as previously described^7^,^34^ and blood sampling was performed on LD7 or LD8. Two hours after lights on, and at least 90 min after attaching the catheter to a tubing filled with heparinised saline (30 IU/ml), a basal sample was collected. 30 min later a second basal sample was collected as a backup. Thirty minutes after collection of the second basal sample, ACTH (0.1 ml to give final stress-related blood concentration of approximately 500 pg/ml^35^) was injected *iv* via the catheter and blood samples collected 5, 15, 30, 60 and 120 min after injection. For each sample 0.25 ml blood were drawn and replaced immediately with sterile 0.9% saline. Due to methodological problems one nulliparous NFD and one nulliparous HFD were removed from the corticosterone analysis.

#### Statistics

Results were analysed using either a one-way or two-way ANOVA with or without repeated measures, as appropriate followed by Bonferroni pairwise *post-hoc* comparisons. Statistical significance was accepted for P < 0.05. Data that fell outside two SD were considered outliers and excluded from statistical analysis. If sphericity was violated P values were adjusted with Huynh-Feldt. Statistical analyses were performed using IBM SPSS (version 21; Chicago, US).

The methods were carried out in accordance with the approved guidelines.

## Additional Information

**How to cite this article**: Perani, C. V. *et al.* High-fat diet prevents adaptive peripartum-associated adrenal gland plasticity and anxiolysis. *Sci. Rep.* **5**, 14821; doi: 10.1038/srep14821 (2015).

## Supplementary Material

Supplementary Information
