# Supplementary material for: High-fat diet prevents adaptive peripartum-associated adrenal gland plasticity and anxiolysis
Source: Sci Rep. 2015 Oct 7;5:14821. doi: 10.1038/srep14821 (PMC4595833; doi:10.1038/srep14821)

TITLE:

High fat diet prevents adaptive peripartum-associated adrenal gland plasticity and anxiolysis

Clara V. Perani, Inga D. Neumann, Stefan O. Reber & David A. Slattery

Supplementary information

Supplementary Methods

Adrenal RNA isolation and qPCR. Right adrenal glands were homogenized in 1 ml TriFast Gold (Peqlab, US) and the aqueous phase obtained through chloroform precipitation was loaded onto a silica membrane (RNeasy Mini kit, Qiagen, Netherlands); RNA was isolated following manufacturer instructions. RNA was reverse transcribed into cDNA using the SuperScript III First-Strand Synthesis System for RT-PCR (Invitrogen, US) and expression levels of Star and Cyp11A1, and of the house keeping gene Glyceraldehyde 3-phosphate dehydrogenase (GAPDH), were measured using SYBR Green (QuantiFast Qiagen, Netherlands). Primer sets containing forward and reverse primers were used (Biomol, germany); after initial activation at 95° C for 5 min, 50 cycles were performed as follows: 95° C for 10 seconds followed by 45 seconds at 60° C. Samples were run in triplicates and normalized to GAPDH.

Supplementary Results

Hormonal and adrenal changes across the estrous cycle. Despite no changes in basal plasma ACTH levels (Supplementary Figure (Fig.) 1a), basal corticosterone varied across the estrous cycle ($F_{2,13}$=19.3, P<0.001; Supplementary Fig. 1b). Specifically, females at proestrous showed higher corticosterone compared with rats at di-/metestrous and estrous (P<0.001 and P<0.01, respectively). Moreover, of all the adrenal parameters assessed only

protein expression of LDLR ($F_{2,16}$=4.97, P=0.021; Supplementary Fig. 1d; Supplementary Table 1) differed across the estrous cycle. Specially, LDLR was increased at estrous compared with di-/metestrous (P<0.05).

Reproductive state did not affect adrenal weight. Adrenal weight did not differ across the peripartum period, but a body side-specific effect ($F_{1,78}$=10.1; P=0.002; Supplementary Fig. 2) on this parameter was observed.

Reproductive state and HFD affect body weight gain. Reproductive state affected body weight gain ($F_{1,25}$=43.1, P<0.001; Supplementary Fig. 3), and an interaction between the diet and day ($F_{10,250}$=6.36, P<0.001). As expected, pregnant animals gained more weight compared with nulliparous (P<0.05), and HFD increased body weight gain in both nulliparous and lactating groups (P<0.05).

Adrenal Star and Cyp11A1 mRNA expression. Neither Star nor Cyp11A1 adrenal mRNA expression was affected by HFD (Supplementary Table 2). We observed a reproductive state effect on Star mRNA ($F_{3,25}$=4.27, P=0.049) although *post hoc* analysis failed to show further differences

HFD did not affect ultrasonic vocalizations or weight gain in the offspring. To assess whether maternal diet affects the offspring, body weight at birth and body weight gain was recorded and the number of vocalizations of one male and one female pup from each litter was measured while the mothers underwent surgical catheter implantation. Specifically, each pup was placed on a petri dish and the number of ultrasonic vocalizations was assessed by a bat detector (22 kHz; BVL, Germany) across 10 minutes. However, neither body weight at birth nor body weight gain was affected. Moreover, 10 minutes pup vocalization assessed after separation from the mother did not differ between the groups.

Supplementary Figures

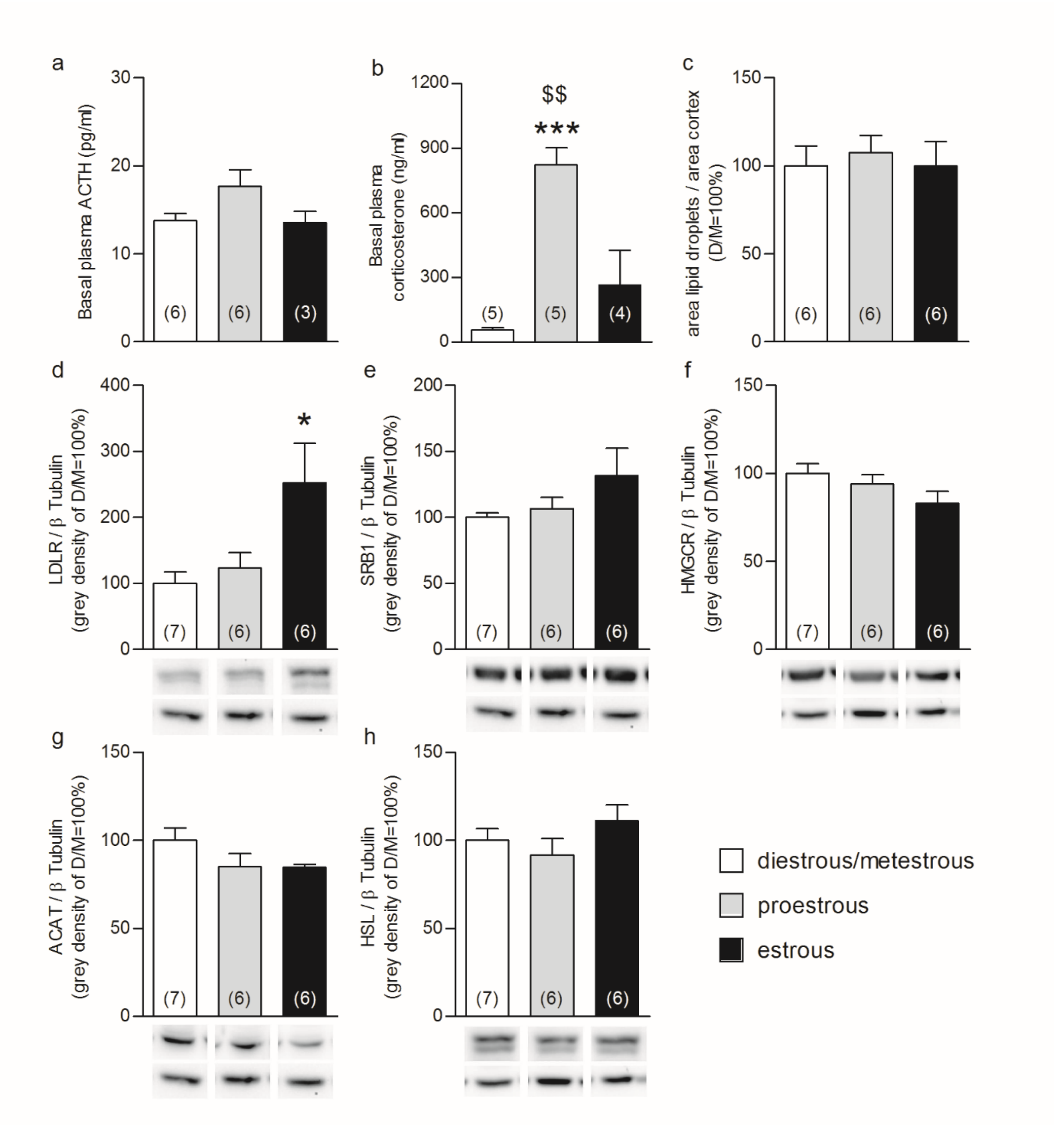

Supplementary Figure 1 Hormone levels and adrenal plasticity across the estrous cycle. Changes in plasma ACTH (a) and corticosterone (b), adrenal cortical lipid droplets (c) and western blot analysis of LDLR (d), SRB1 (e), HMGCR (f), ACAT (g) and HSL (h) in nulliparous rats at diestrous/metestrous, proestrous, and estrous are represented. Lipid droplets and protein measures are expressed relative to rats at diestrous/metestrous set to 100%. All protein bands were normalized to β tubulin level in the same membrane.

Representative blots are reported below the corresponding bars of the protein of interest (upper band) together with the loading control (lower band). Data represent mean + SEM (numbers in parenthesis indicate group sizes). *** P<0.001 and * P<0.05 vs. diestrous/metestrous; $$ P<0.01 vs. estrous females. Statistical significance was determined using a one-way ANOVA followed by Bonferroni *post-hoc* test.

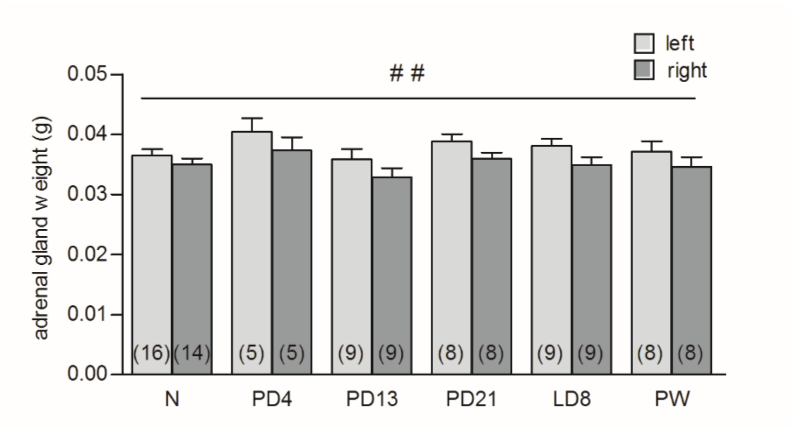

Supplementary Figure 2 Adrenal gland weight. Body side and status effects on absolute adrenal gland weight were assessed in nulliparous (N) rats, at pregnancy day (PD) 4, PD13, PD21, LD8, and 4 weeks post weaning (PW). Data represent mean + SEM (numbers in parenthesis indicate group sizes). ## P <0.01 main ANOVA effect of body side. Statistical significance was determined using a two-way ANOVA.

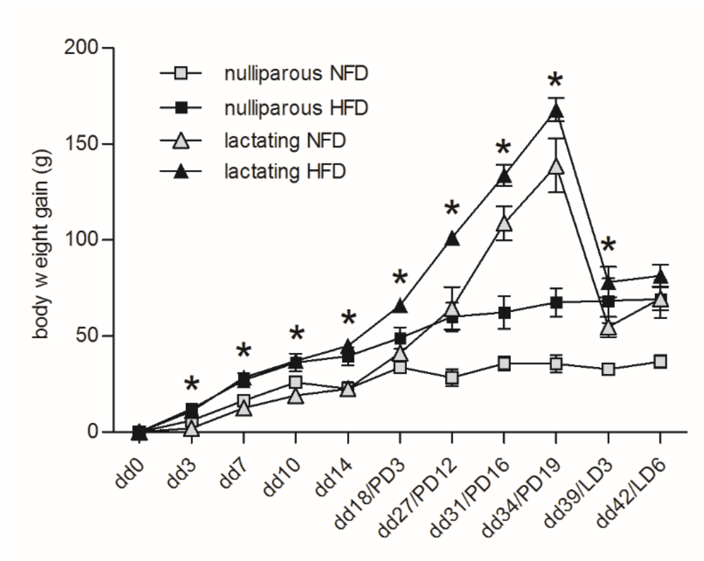

Supplementary Figure 3 Body weight gain before and after mating in normal fat diet (NFD) and high-fat diet (HFD) fed rats. Diet day (dd); pregnancy day (PD); lactation day (LD). Data represent mean +/- SEM. * P <0.05 vs. respective HFD group. Statistical significance was determined using a two-way ANOVA for repeated measures followed by Bonferroni *post-hoc* test.

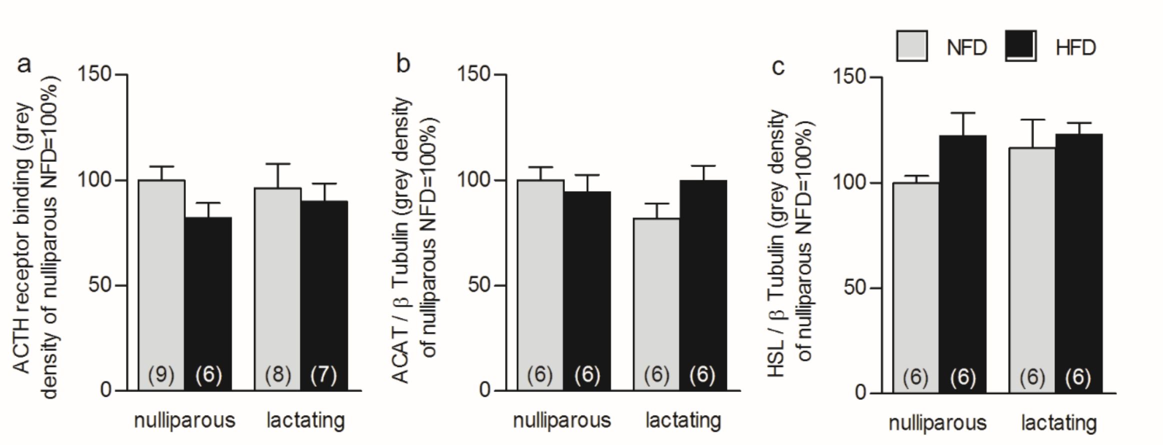

Supplementary Figure 4 High-fat diet (HFD) did not affect adrenal ACTH-receptor binding and ACAT and HSL protein expression. Adrenal ACTH-receptor binding (a) measured via receptor autoradiography and western blot analysis of ACAT and HSL protein expression (b and c) in nulliparous and lactating normal fat diet (NFD) and HFD rats are represented. Protein measures were normalized to β tubulin in the same membrane and are expressed relative to virgin NFD set to 100%. None of the parameters was affected. Data represent mean + SEM (numbers in parenthesis indicate group sizes). Statistical significance was determined using a two-way ANOVA.

Supplementary Tables

	Left	Right
LDLR protein expression (grey density of left measurement=100%)	100±40.2	115.6±48.2
SRB1 protein expression (grey density of left measurement=100%)	100±19.4	103.7±22.2
HMGCR protein expression (grey density of left measurement=100%)	100±14.1	95.8±11.8
ACAT protein expression (grey density of left measurement=100%)	100±19.5	105.3±24.8
HSL protein expression (grey density of left measurement=100%)	100±29.3	103.9±22.9
lipid droplets (area lipid droplets/area cortex of left adrenals=100%)	100±25.7	73.4±15.7

Supplementary Table 1 The expression of adrenal lipid droplets and enzymes important for cholesterol supply for steroidogenesis are not body side-specific in nulliparous rats. Left and right differences in adrenal protein levels were assessed in four nulliparous rats at diestrous/metestrous, four at proestrous and four at estrous (n=12); grey density of left measurements are set to 100 % and all protein bands were normalized to β Tubulin levels in the same membrane. Side differences in adrenal lipid droplets were assessed in two nulliparous at diestrous/metestrous, two at proestrous and two at estrous (n=6) and the ratio between the lipid droplets area and the cortex area is expressed, setting the left measures to 100 %. Statistical significance was determined using two-tailed Student’s tests.

	Virgin NFD	Lactating NFD	Virgin HFD	Lactating HFD
Star relative mRNA levels (Virgin NFD=1)	1±0.21 (8)	1.8±1.59 (7)	0.74±0.21 (7)	1.26±0.71 (7)
Cyp11A1 relative mRNA levels (Virgin NFD=1)	1±0.58 (8)	0.77±0.45 (8)	1.2±0.99 (8)	1.28±0.94 (6)

Supplementary Table 2 Adrenal Star and Cyp11A1 mRNA is not affected by high-fat diet (HFD) feeding. Adrenal Star and Cyp11A1 mRNA expression was assessed *via* qPCR in virgin and lactating rats fed on a normal fat diet (NFD) and HFD. The reproductive state affected Star expression although *post hoc* analysis failed to reveal further significant differences. Data are expressed relative to virgin NFD set to 1 ± standard deviation (numbers in parenthesis indicate group sizes). Samples were run in triplicates and Star and Cyp11A1 measures normalized for GAPDH. Statistical significance was determined using a two-way ANOVA.

Full unedited gels for Figure 1d

Gel 1

LDLR

β Tubulin

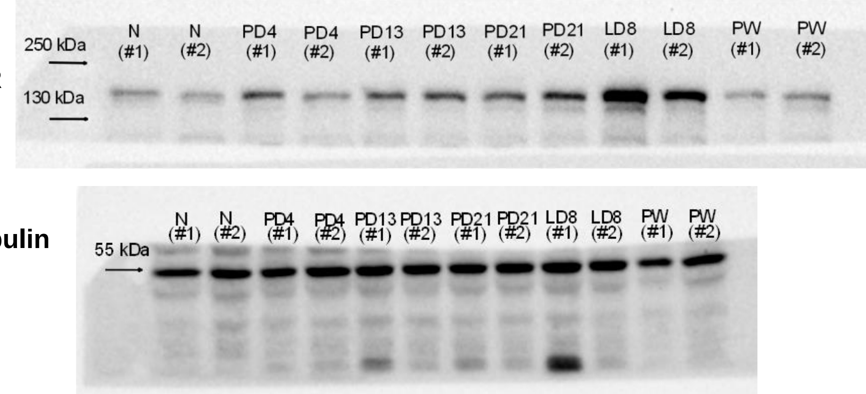

Duplicate gel 1

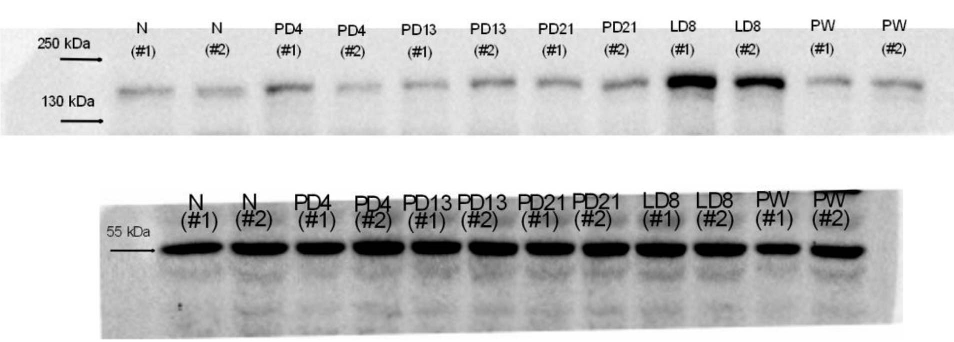

Gel 2

LDLR

β Tubulin

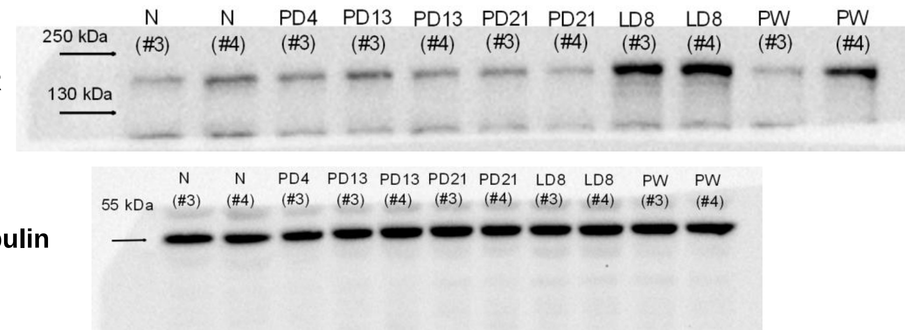

Duplicate gel 2

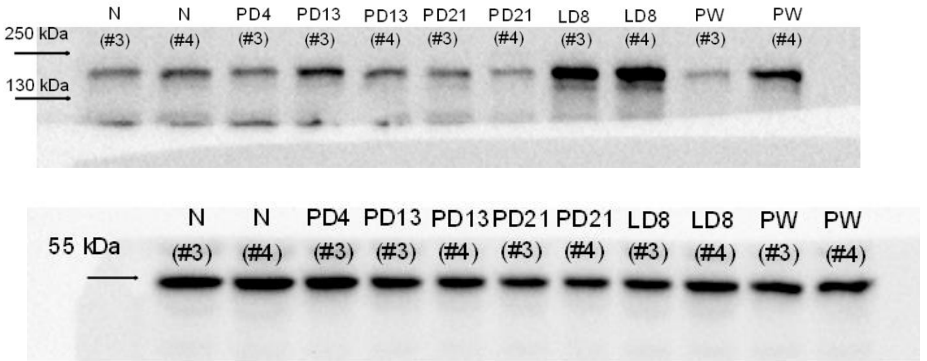

Gel 3

LDLR

β Tubulin

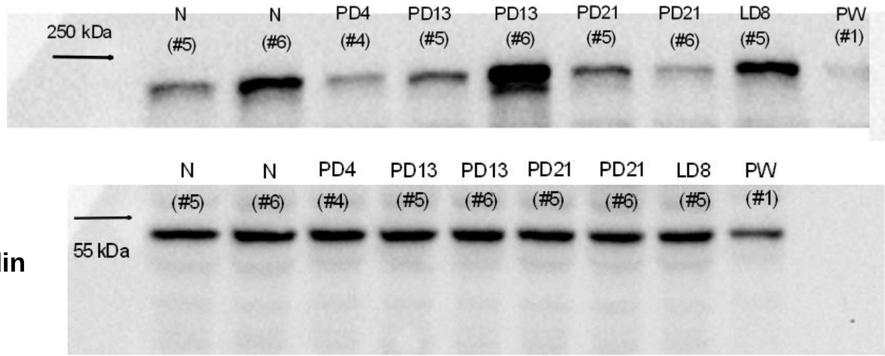

Duplicate gel 3

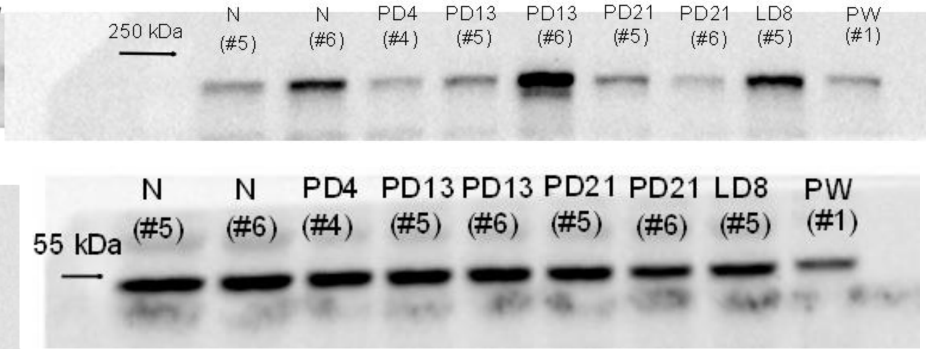

Full unedited gels for Figure 1e

Gel 1

SRB1

β Tubulin

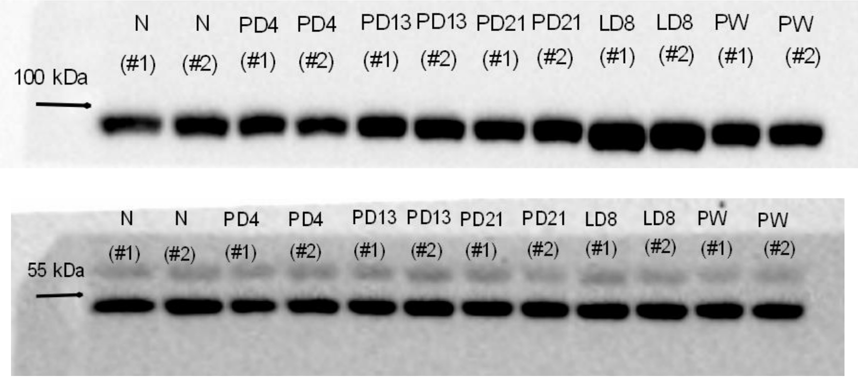

Duplicate gel 1

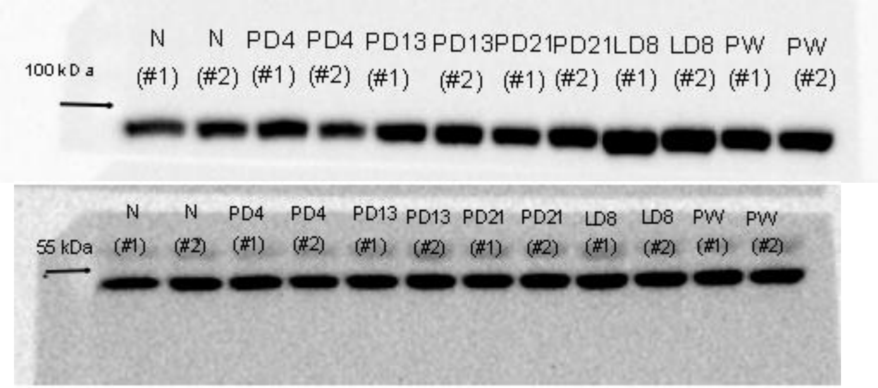

Gel 2

SRB1

β Tubulin

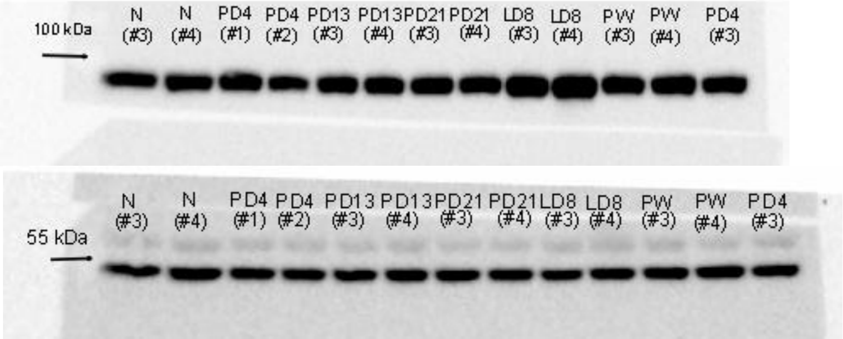

Duplicate gel 2

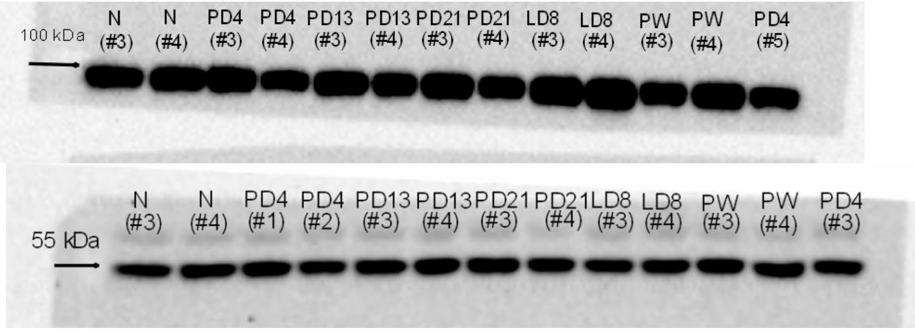

Gel 3

SRB1

β Tubulin

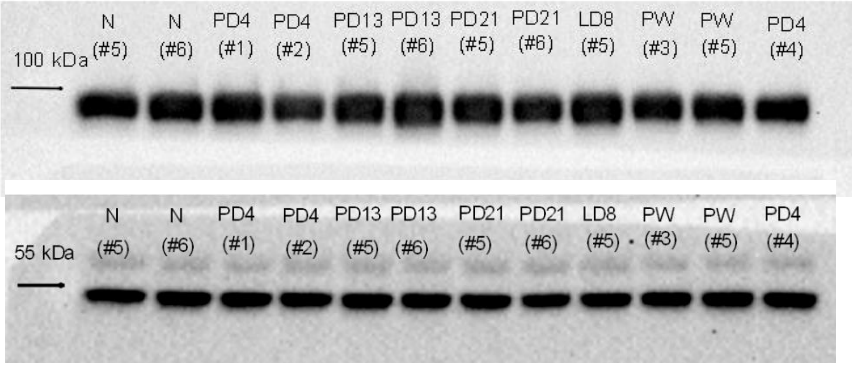

Duplicate gel 3

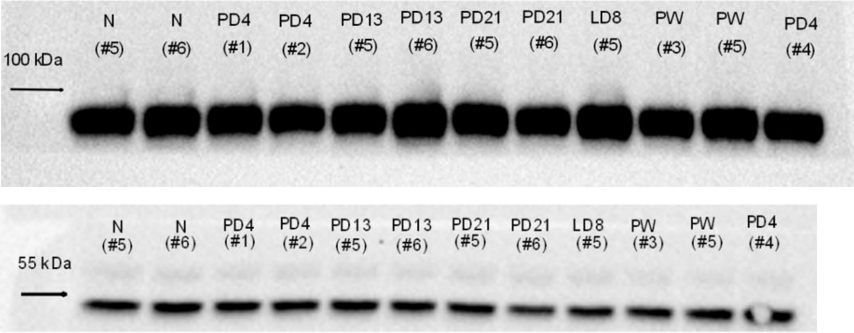

Full unedited gels for Figure 1f

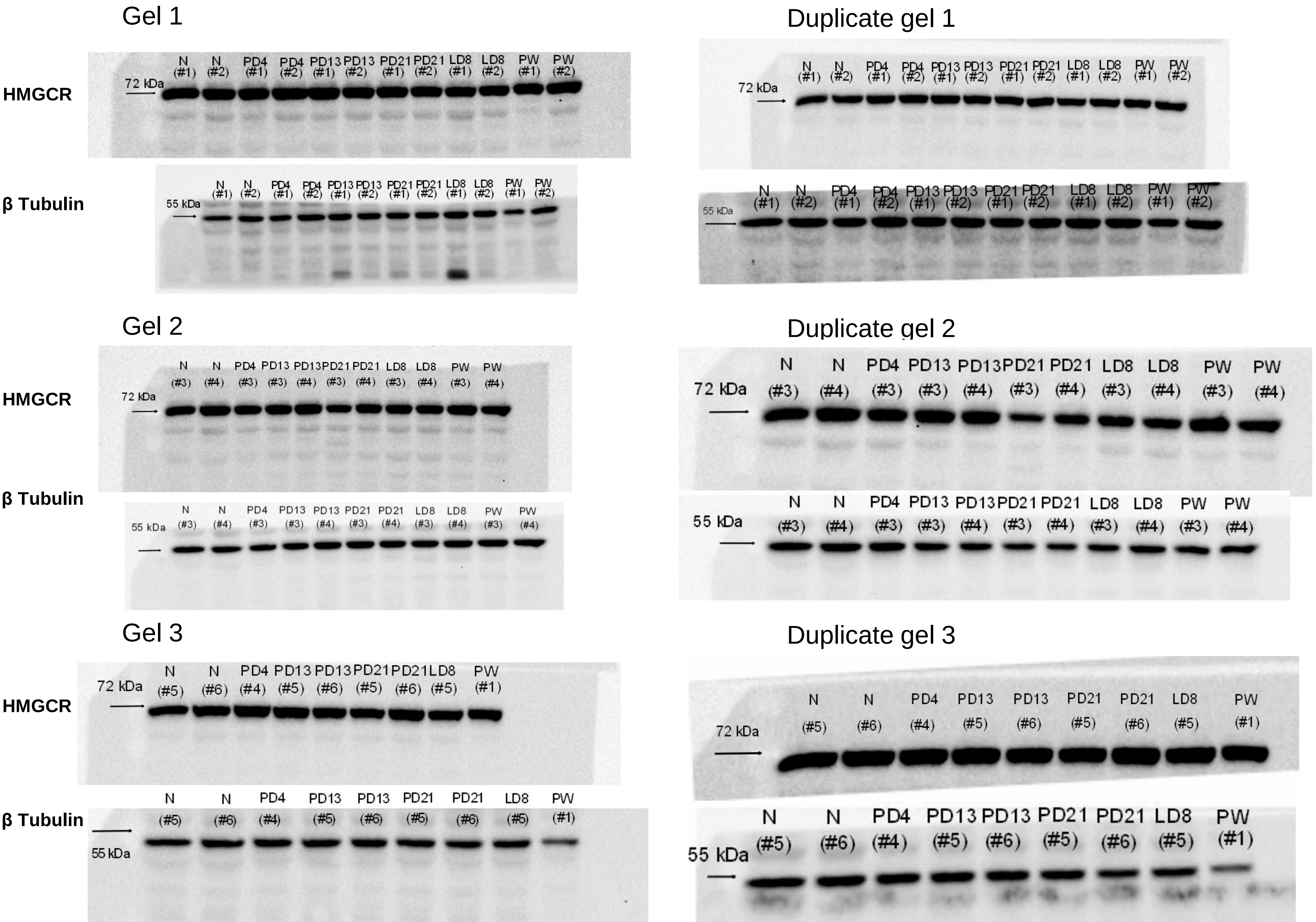

Full unedited gels for Figure 1g

Gel 1

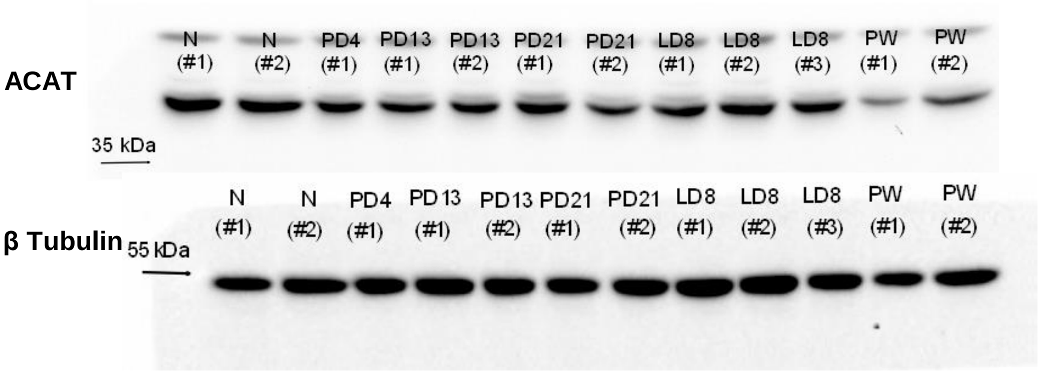

Duplicate gel 1

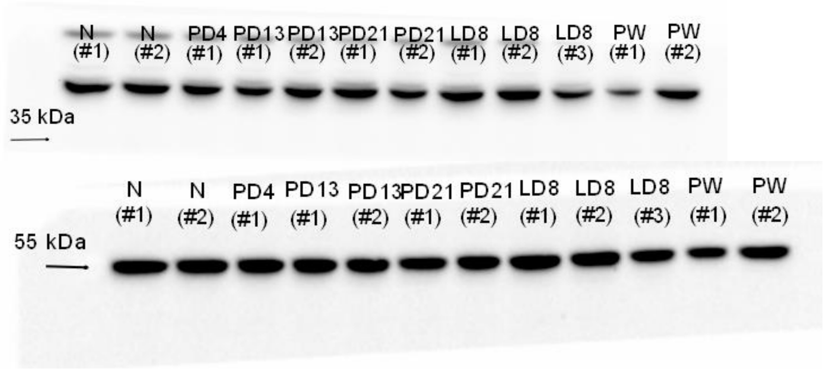

Gel 2

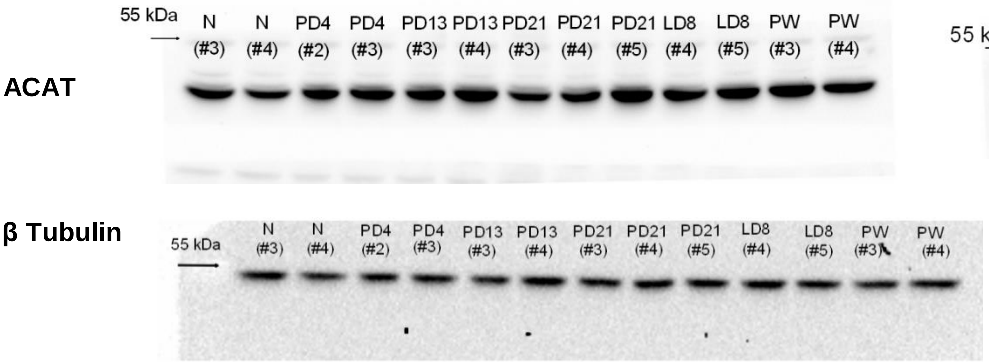

Duplicate gel 2

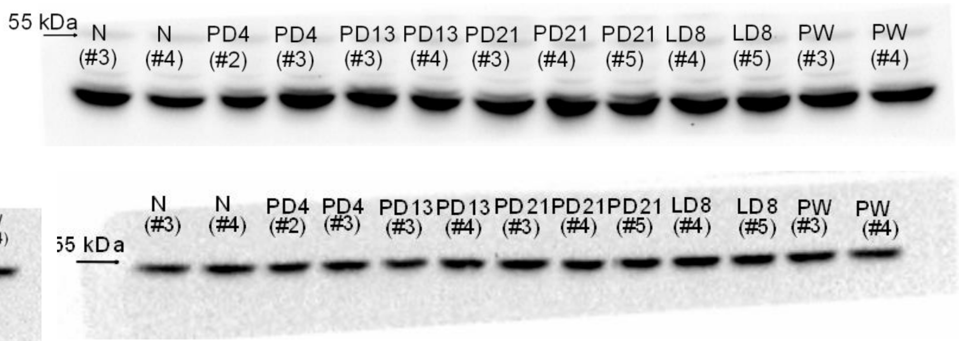

Gel 3

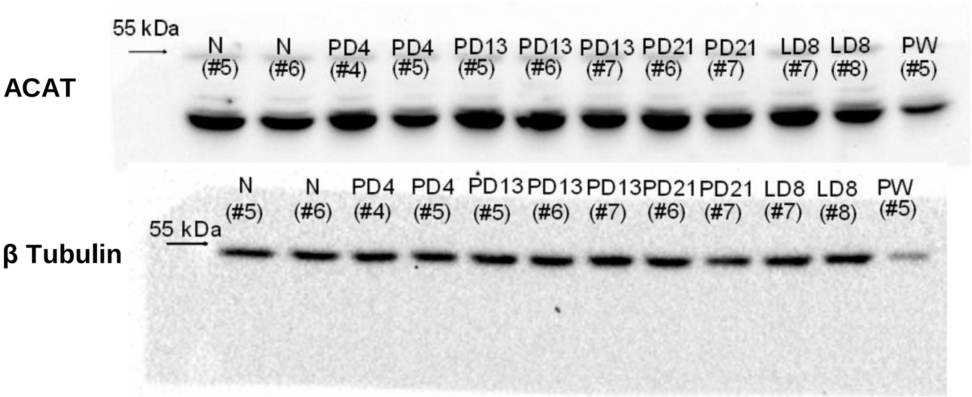

Duplicate gel 3

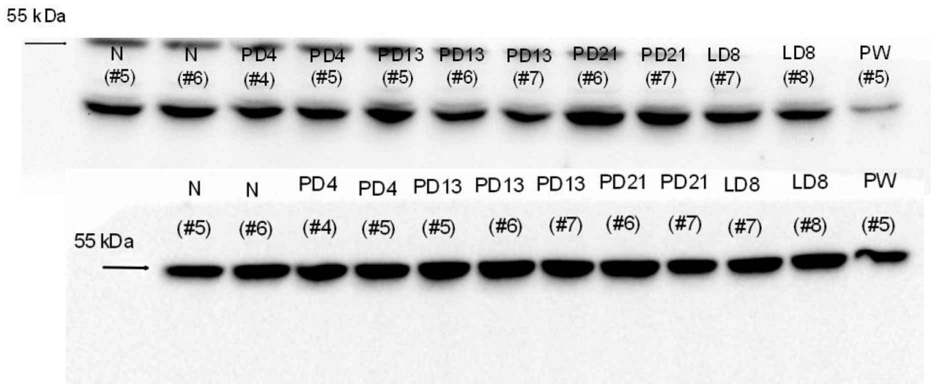

Full unedited gels for Figure 1h

Gel 1

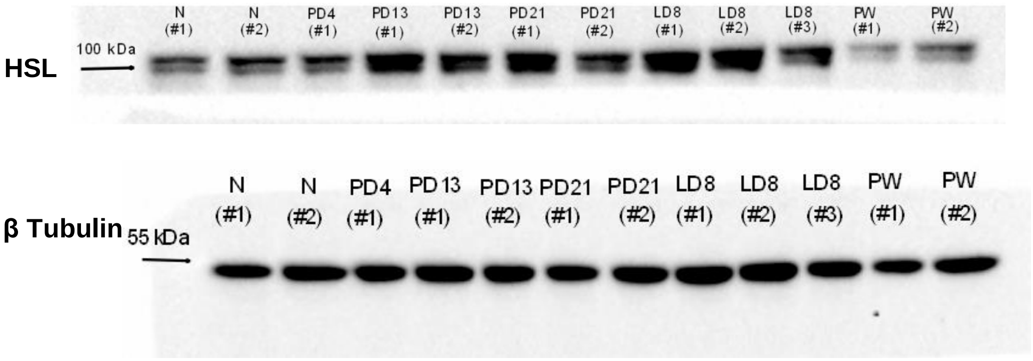

Duplicate gel 1

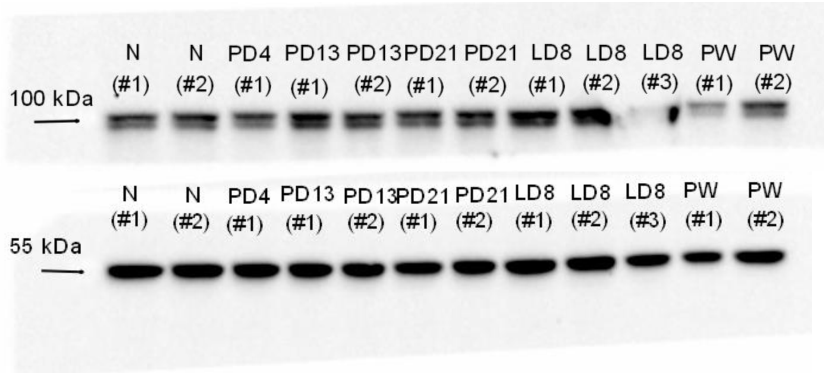

Gel 2

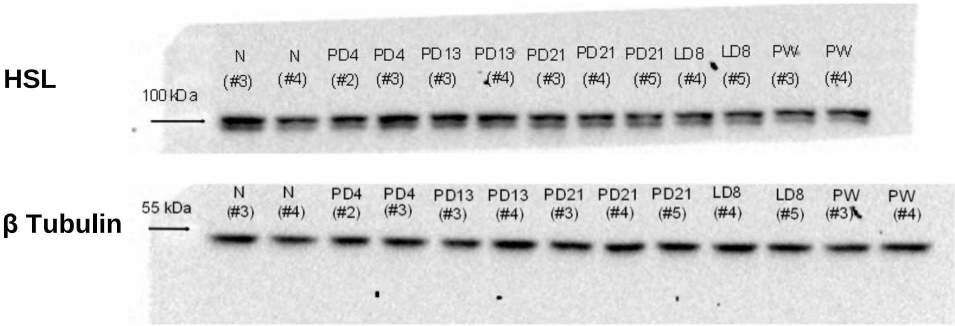

Duplicate gel 2

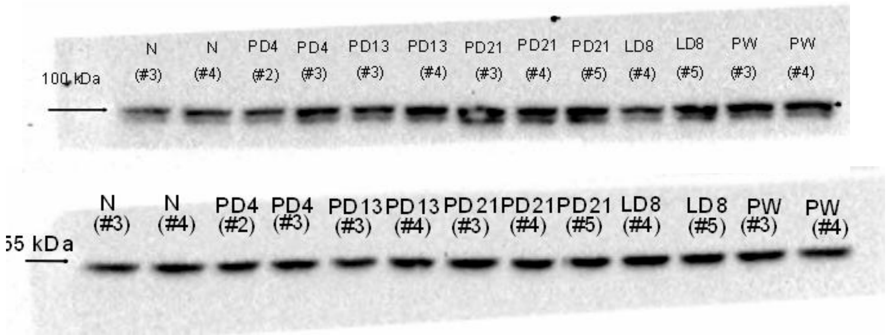

Gel 3

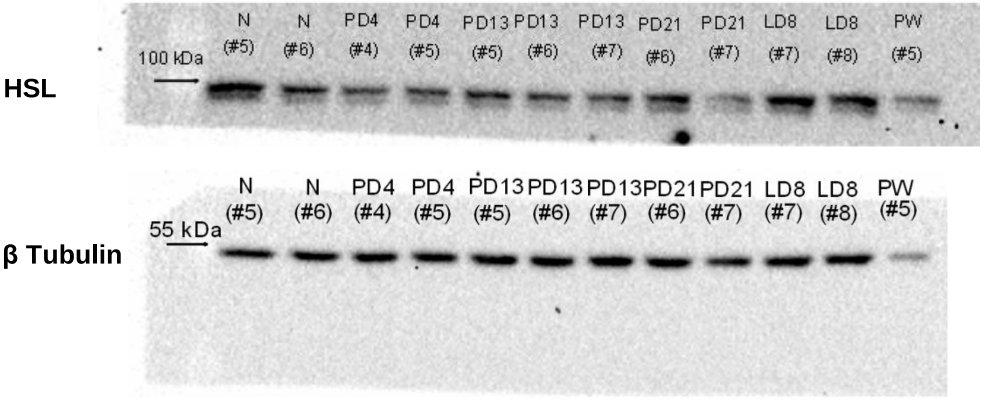

Duplicate gel 3

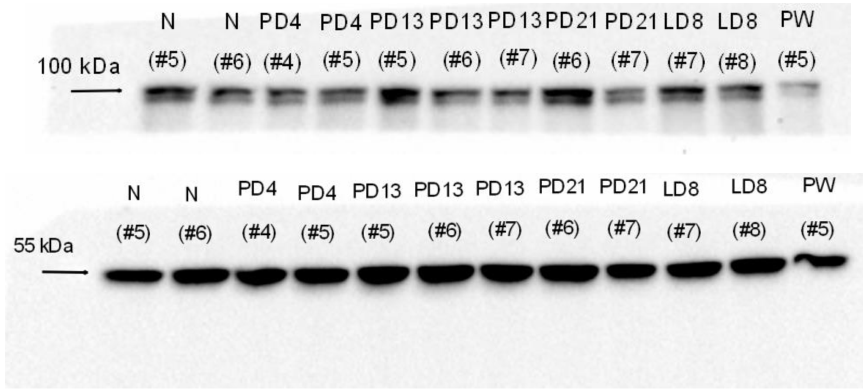

Full unedited gels for Figure 4a

Gel 1

LDLR

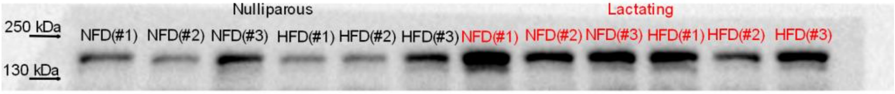

β Tubulin

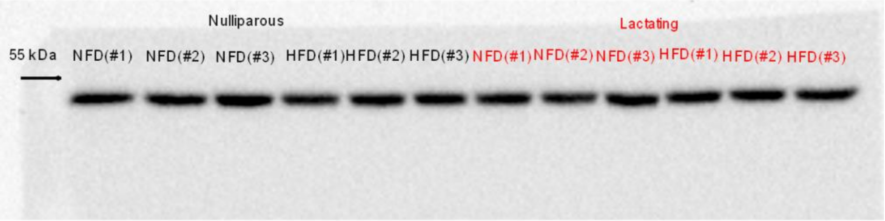

Duplicate gel 1

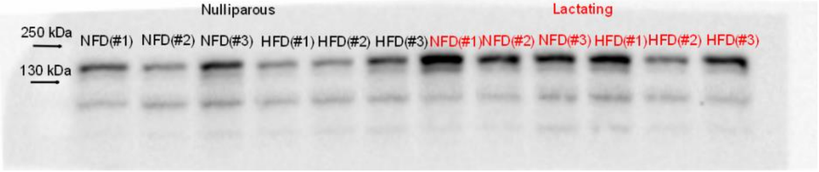

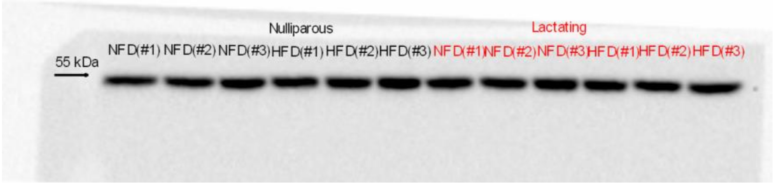

Gel 2

LDLR

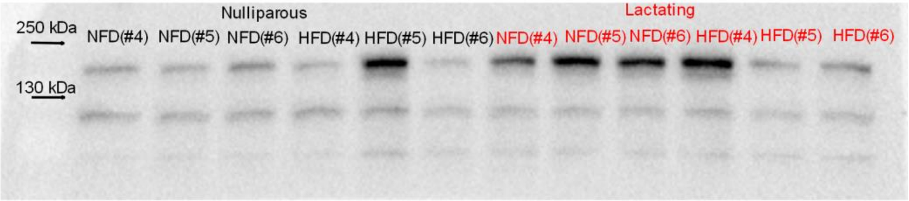

β Tubulin

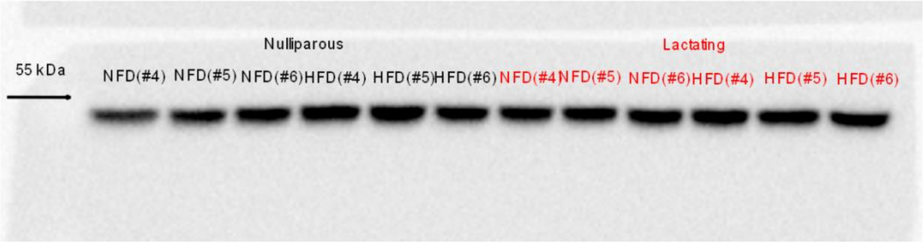

Duplicate gel 2

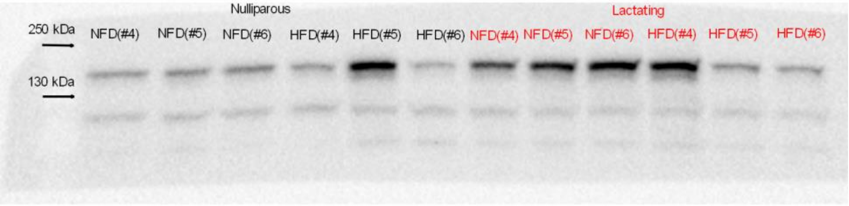

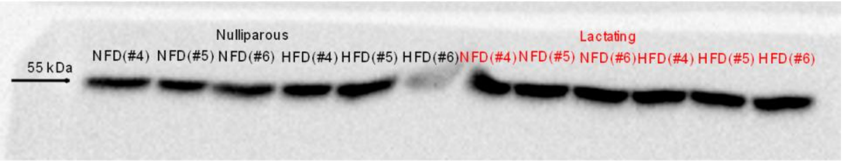

Full unedited gels for Figure 4b

Gel 1

Duplicate gel 1

SRB1

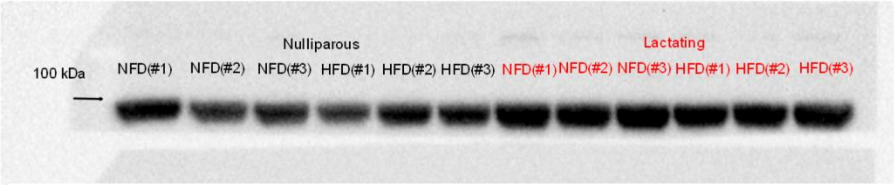

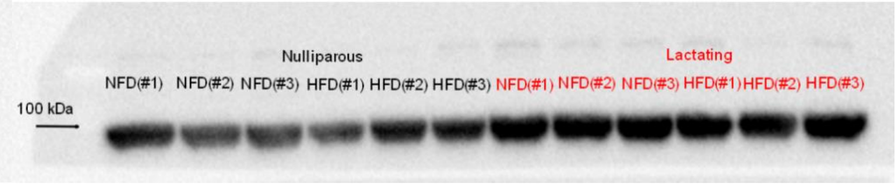

β Tubulin

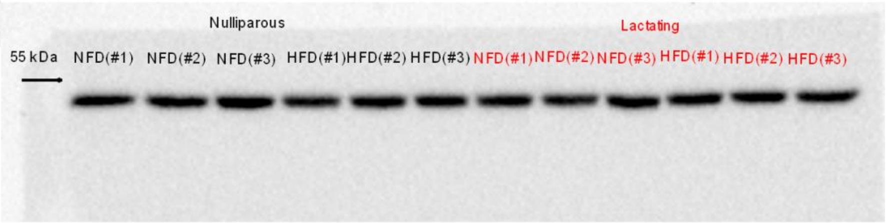

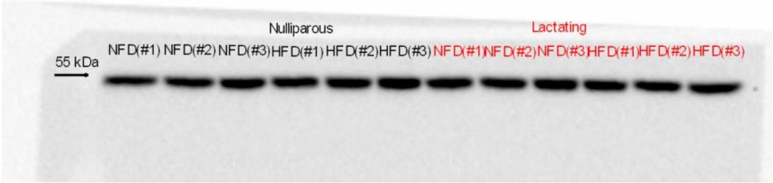

Gel 2

Duplicate gel 2

SRB1

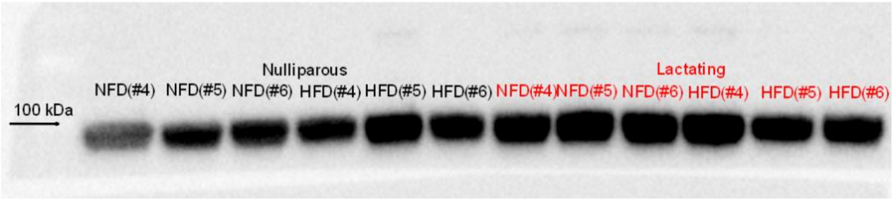

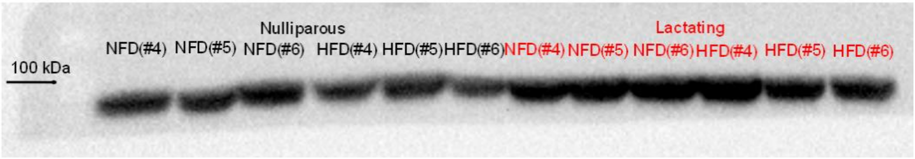

β Tubulin

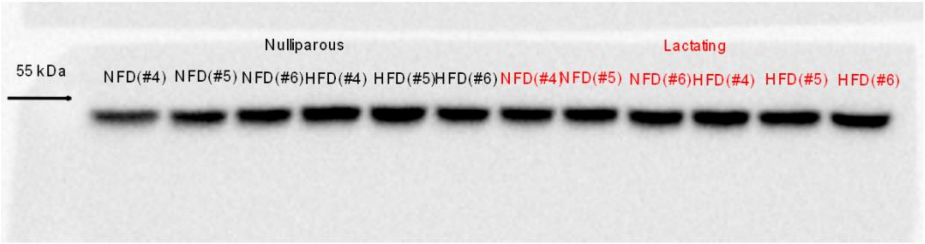

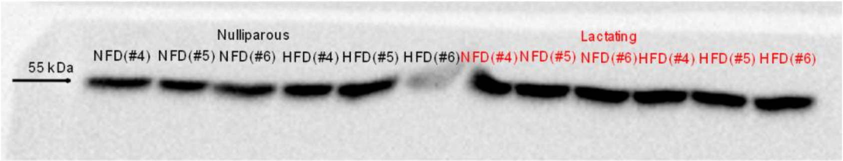

Full unedited gels for Figure 4c

Gel 1

Duplicate gel 1

HMGCR

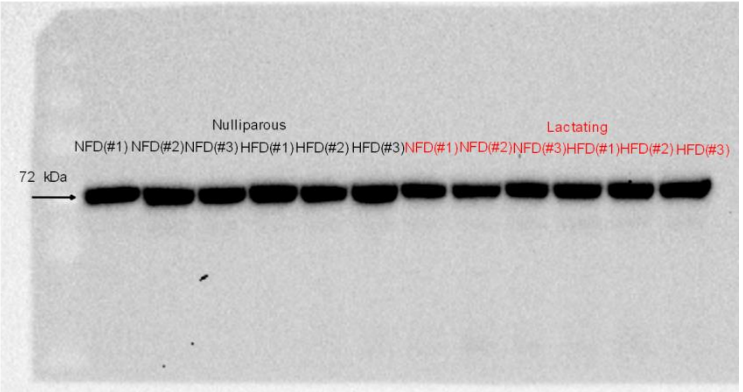

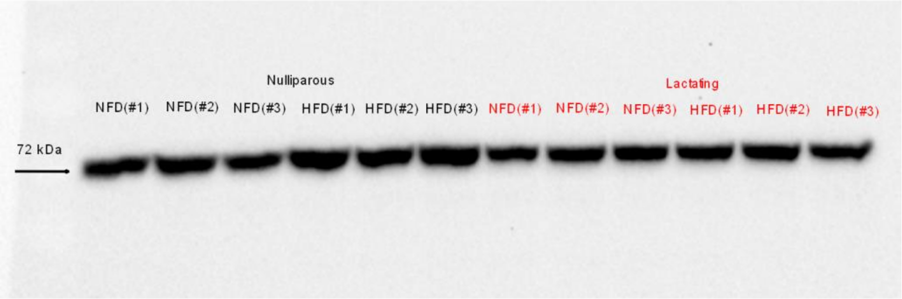

β Tubulin

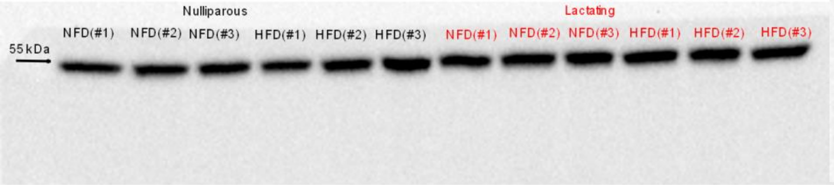

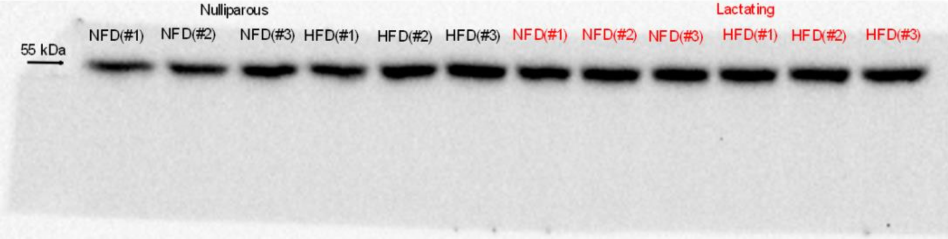

Gel 2

Duplicate gel 2

HMGCR

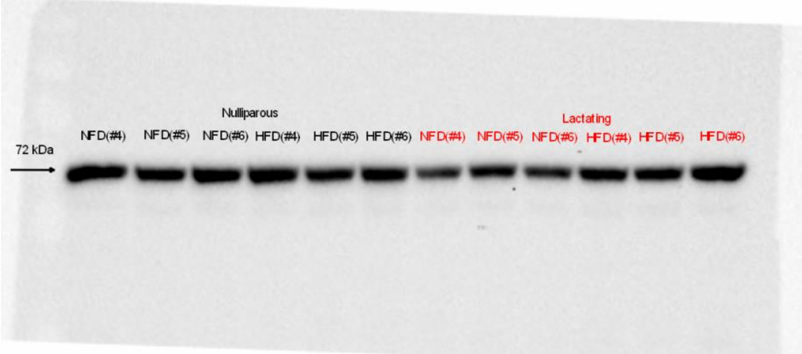

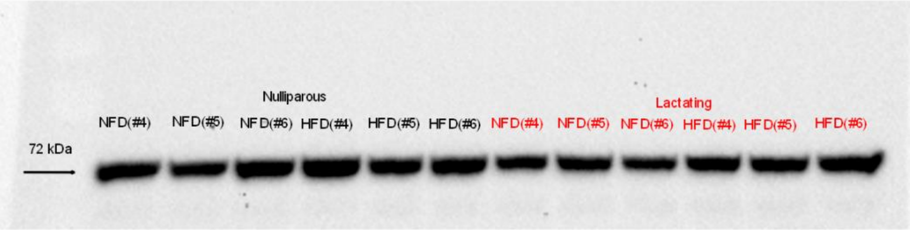

β Tubulin

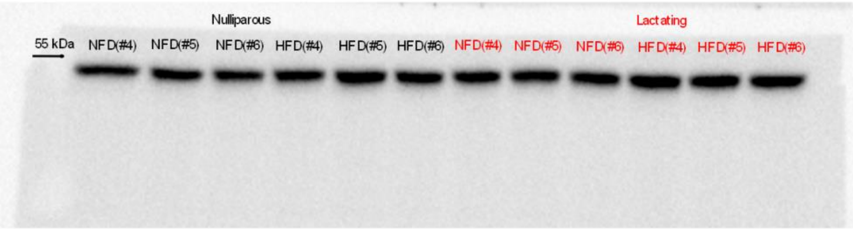

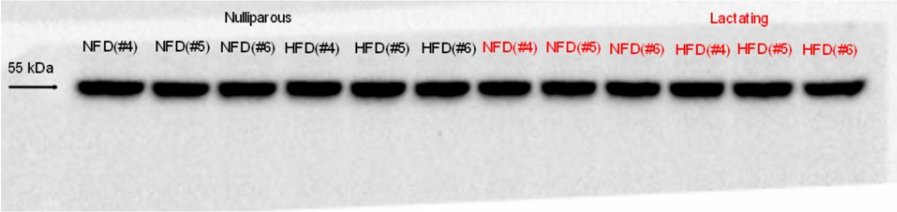

Supplement: Supplementary Information [file srep14821-s1.pdf]
